# Supplementary material for: Multi‐dataset identification of innovative feature genes and molecular mechanisms in keratoconus
Source: J Cell Mol Med. 2024 Sep 19;28(18):e70079. doi: 10.1111/jcmm.70079 (PMC11412914; doi:10.1111/jcmm.70079)
Supplement: Supplementary file 2 — Material S2. [file JCMM-28-e70079-s002.zip › 7.GSEA analysis/3.DBNDD1/gsea_report_for_h_1678852837355.html]

Report for h 1678852837355 [GSEA]

| GS  follow link to MSigDB | GS DETAILS | SIZE | ES | NES | NOM p-val | FDR q-val | FWER p-val | RANK AT MAX | LEADING EDGE || 1 | KEGG\_PENTOSE\_AND\_GLUCURONATE\_INTERCONVERSIONS | Details ... | 20 | 0.57 | 1.55 | 0.043 | 0.923 | 0.691 | 6027 | tags=35%, list=17%, signal=42% |
| 2 | KEGG\_ASCORBATE\_AND\_ALDARATE\_METABOLISM | Details ... | 17 | 0.52 | 1.42 | 0.078 | 1.000 | 0.871 | 2660 | tags=24%, list=8%, signal=25% |
| 3 | KEGG\_PORPHYRIN\_AND\_CHLOROPHYLL\_METABOLISM | Details ... | 32 | 0.43 | 1.39 | 0.082 | 0.903 | 0.897 | 6362 | tags=22%, list=18%, signal=27% |
| 4 | KEGG\_TERPENOID\_BACKBONE\_BIOSYNTHESIS | Details ... | 15 | 0.52 | 1.37 | 0.137 | 0.751 | 0.909 | 11996 | tags=53%, list=35%, signal=81% |
| 5 | KEGG\_GLYOXYLATE\_AND\_DICARBOXYLATE\_METABOLISM | Details ... | 16 | 0.49 | 1.36 | 0.132 | 0.646 | 0.920 | 6612 | tags=25%, list=19%, signal=31% |
| 6 | KEGG\_RNA\_DEGRADATION | Details ... | 51 | 0.26 | 1.32 | 0.048 | 0.665 | 0.943 | 25700 | tags=100%, list=74%, signal=384% |
| 7 | KEGG\_RIBOSOME | Details ... | 85 | 0.30 | 1.11 | 0.312 | 1.000 | 0.996 | 20568 | tags=96%, list=59%, signal=236% |
| 8 | KEGG\_PHENYLALANINE\_METABOLISM | Details ... | 17 | 0.37 | 1.01 | 0.457 | 1.000 | 1.000 | 5309 | tags=29%, list=15%, signal=35% |
| 9 | KEGG\_GLYCOSAMINOGLYCAN\_DEGRADATION | Details ... | 21 | 0.32 | 1.00 | 0.447 | 1.000 | 1.000 | 14473 | tags=62%, list=42%, signal=106% |
| 10 | KEGG\_NEUROACTIVE\_LIGAND\_RECEPTOR\_INTERACTION | Details ... | 270 | 0.27 | 0.99 | 0.457 | 1.000 | 1.000 | 6350 | tags=26%, list=18%, signal=32% |
| 11 | KEGG\_PROTEIN\_EXPORT | Details ... | 22 | 0.28 | 0.92 | 0.599 | 1.000 | 1.000 | 24851 | tags=100%, list=72%, signal=351% |
| 12 | KEGG\_SPLICEOSOME | Details ... | 125 | 0.21 | 0.90 | 0.537 | 1.000 | 1.000 | 25270 | tags=98%, list=73%, signal=360% |
| 13 | KEGG\_CYSTEINE\_AND\_METHIONINE\_METABOLISM | Details ... | 34 | 0.20 | 0.89 | 0.636 | 1.000 | 1.000 | 10641 | tags=29%, list=31%, signal=42% |
| 14 | KEGG\_PARKINSONS\_DISEASE | Details ... | 98 | 0.21 | 0.88 | 0.562 | 1.000 | 1.000 | 16011 | tags=52%, list=46%, signal=96% |
| 15 | KEGG\_GLYCINE\_SERINE\_AND\_THREONINE\_METABOLISM | Details ... | 31 | 0.26 | 0.88 | 0.686 | 1.000 | 1.000 | 5079 | tags=23%, list=15%, signal=26% |
| 16 | KEGG\_PEROXISOME | Details ... | 78 | 0.23 | 0.87 | 0.621 | 1.000 | 1.000 | 11488 | tags=35%, list=33%, signal=52% |
| 17 | KEGG\_ARGININE\_AND\_PROLINE\_METABOLISM | Details ... | 52 | 0.24 | 0.86 | 0.677 | 1.000 | 1.000 | 2879 | tags=10%, list=8%, signal=10% |
| 18 | KEGG\_COLORECTAL\_CANCER | Details ... | 62 | 0.21 | 0.85 | 0.667 | 1.000 | 1.000 | 1824 | tags=5%, list=5%, signal=5% |
| 19 | KEGG\_ABC\_TRANSPORTERS | Details ... | 44 | 0.28 | 0.84 | 0.654 | 1.000 | 1.000 | 7468 | tags=30%, list=22%, signal=38% |
| 20 | KEGG\_OLFACTORY\_TRANSDUCTION | Details ... | 372 | 0.25 | 0.81 | 0.975 | 1.000 | 1.000 | 5623 | tags=26%, list=16%, signal=31% |
| 21 | KEGG\_PANCREATIC\_CANCER | Details ... | 69 | 0.18 | 0.79 | 0.729 | 1.000 | 1.000 | 1824 | tags=4%, list=5%, signal=5% |
| 22 | KEGG\_RNA\_POLYMERASE | Details ... | 27 | 0.23 | 0.76 | 0.862 | 1.000 | 1.000 | 26624 | tags=100%, list=77%, signal=428% |
| 23 | KEGG\_DRUG\_METABOLISM\_OTHER\_ENZYMES | Details ... | 42 | 0.23 | 0.74 | 0.888 | 1.000 | 1.000 | 2660 | tags=10%, list=8%, signal=10% |
| 24 | KEGG\_GLYCEROLIPID\_METABOLISM | Details ... | 43 | 0.21 | 0.74 | 0.887 | 1.000 | 1.000 | 11468 | tags=47%, list=33%, signal=69% |
| 25 | KEGG\_OXIDATIVE\_PHOSPHORYLATION | Details ... | 98 | 0.17 | 0.72 | 0.776 | 1.000 | 1.000 | 17227 | tags=65%, list=50%, signal=129% |
| 26 | KEGG\_NON\_SMALL\_CELL\_LUNG\_CANCER | Details ... | 54 | 0.16 | 0.72 | 0.848 | 1.000 | 1.000 | 35 | tags=2%, list=0%, signal=2% |
| 27 | KEGG\_ENDOMETRIAL\_CANCER | Details ... | 52 | 0.18 | 0.71 | 0.819 | 1.000 | 1.000 | 35 | tags=2%, list=0%, signal=2% |
| 28 | KEGG\_BUTANOATE\_METABOLISM | Details ... | 34 | 0.24 | 0.71 | 0.840 | 1.000 | 1.000 | 606 | tags=6%, list=2%, signal=6% |
| 29 | KEGG\_OOCYTE\_MEIOSIS | Details ... | 111 | 0.14 | 0.70 | 0.899 | 1.000 | 1.000 | 8784 | tags=19%, list=25%, signal=25% |
| 30 | KEGG\_GLYCOSAMINOGLYCAN\_BIOSYNTHESIS\_HEPARAN\_SULFATE | Details ... | 26 | 0.21 | 0.70 | 0.908 | 1.000 | 1.000 | 7740 | tags=19%, list=22%, signal=25% |
| 31 | KEGG\_AMINOACYL\_TRNA\_BIOSYNTHESIS | Details ... | 22 | 0.22 | 0.66 | 0.920 | 1.000 | 1.000 | 27116 | tags=100%, list=78%, signal=456% |
| 32 | KEGG\_STARCH\_AND\_SUCROSE\_METABOLISM | Details ... | 39 | 0.22 | 0.65 | 0.916 | 1.000 | 1.000 | 4858 | tags=15%, list=14%, signal=18% |
| 33 | KEGG\_APOPTOSIS | Details ... | 87 | 0.16 | 0.62 | 0.966 | 1.000 | 1.000 | 2920 | tags=6%, list=8%, signal=6% |
| 34 | KEGG\_NICOTINATE\_AND\_NICOTINAMIDE\_METABOLISM | Details ... | 24 | 0.23 | 0.60 | 0.895 | 1.000 | 1.000 | 9797 | tags=38%, list=28%, signal=52% |
| 35 | KEGG\_GLYCOSYLPHOSPHATIDYLINOSITOL\_GPI\_ANCHOR\_BIOSYNTHESIS | Details ... | 25 | 0.19 | 0.60 | 0.879 | 1.000 | 1.000 | 27993 | tags=100%, list=81%, signal=515% |
| 36 | KEGG\_PRIMARY\_IMMUNODEFICIENCY | Details ... | 35 | 0.20 | 0.56 | 0.994 | 1.000 | 1.000 | 9218 | tags=31%, list=27%, signal=43% |
| 37 | KEGG\_VALINE\_LEUCINE\_AND\_ISOLEUCINE\_DEGRADATION | Details ... | 43 | 0.15 | 0.54 | 0.960 | 0.977 | 1.000 | 275 | tags=2%, list=1%, signal=2% |
Table: Gene sets enriched in phenotype **h (8 samples) **[plain text format]****
